# Sensitivity of spontaneous abortion classification to hierarchical algorithm assumptions in US commercial claims data, 2018–2024

**DOI:** 10.1371/journal.pone.0361330

**Published:** 2026-10-28

**Authors:** Megan Fuerst, Kaitlin Schrote, Maria I. Rodriguez

**Affiliations:** 1 Division of Complex Family Planning, Oregon Health & Science University, Portland, Oregon, United States of America; 2 Center for Reproductive Health Equity, Department of Obstetrics and Gynecology, Oregon Health & Science University, Portland, Oregon, United States of America; ICAR-Indian Veterinary Research Institute: Indian Veterinary Research Institute, INDIA

## Abstract

**Background:**

Identifying spontaneous abortion (SAB) and induced abortion (IAB) in administrative data compared to other pregnancy outcomes is challenging due to shared billing codes and lack of reimbursement by many insurers.

**Methods:**

We performed a retrospective cohort study using the Merative MarketScan Commercial Database from 2018–2024. We adapted a previously published hierarchical algorithm to reduce the number of unknown or ambiguous pregnancy outcomes. We then tested the assumptions of the hierarchy by alternating the order of the diagnosis, procedural, and diagnosis-related group (DRG) codes within the algorithm for SAB and IAB.

**Results:**

Our dataset included 2,418,986 pregnancy episodes. Our modified algorithm improved the sensitivity for SAB, identifying a 17.5% incidence compared to 14.9% for previously published algorithms and reduced unclassified outcomes from 17.4% to 13.4%. Prioritizing IAB in the algorithm hierarchy over SAB identified an additional 8,623 IAB cases. However, IAB identification remained low at less than 5% of pregnancy episodes.

**Conclusions:**

Following the *Dobbs* decision, it is critical that researchers accurately identify early pregnancy claims and remain transparent in their methodology. Our algorithm improved sensitivity for SAB and reduced unknown outcomes. However, claims-based analyses may systematically undercount IAB, with implications for research-informed reproductive health policy.

## Introduction

Spontaneous abortion (SAB), defined as pregnancy loss before 20 weeks gestation, is the most common complication of early pregnancy [1]. Over 1 million SAB occur annually in the United States (US), significantly outnumbering other non-live birth outcomes [2]. Despite its prevalence, identifying the true incidence of SAB in administrative data is a challenge. SAB are not always seen within the health system, and when they are, may be conflated with induced abortions (IAB). SAB and IAB frequently use the same medications (mifepristone and misoprostol) and the same procedures (Dilation and evacuation) [3]. When these outcomes are conflated, it can affect reproductive health policy more broadly due to misrepresentative data.

Classifying pregnancy outcomes in claims data is complicated by the fact that pregnancy episodes frequently contain codes for more than one outcome type. Individual diagnostic codes are unreliable: a validation study of ICD-9 code 632 for missed abortion found a sensitivity of only 42%, with many cases coded under other diagnoses [4]. Combinations of diagnosis, Diagnosis Related Group (DRG) and procedure codes, are instead used to improve accuracy. Hierarchical algorithms assign each pregnancy a single outcome when multiple outcome types are present [5]. When codes for multiple outcomes are present in a single pregnancy episode, the pregnancy outcome can either be confirmed with other data sources, such as linking it with an infant birth record, or the pregnancy outcome that occurs first in the hierarchy is prioritized. Algorithms differ in their hierarchy assumptions, particularly whether SAB or IAB takes precedence when both are present, and none have tested the effect of these assumptions [6–8].

Zhu et al. developed and validated claims-based algorithms for non-live birth outcomes in Medicaid data, reporting positive predictive values of 79–87 percent for SAB, but relied on ICD-9 codes and excluded rather than resolved cases with overlapping outcome codes [9]. Moll et al. validated pregnancy outcome classification against electronic medical records in the ICD-10 era using a linked MarketScan dataset, but only 9.9 percent of pregnancies had linked clinical records available for adjudication, and their hierarchy prioritized IAB over SAB [7]. The algorithm developed by Ailes et al. provides the most comprehensive hierarchical framework for pregnancy outcome identification across the ICD-9 and ICD-10 eras in MarketScan, but reverses this assumption, prioritizing SAB over IAB when both codes are present [5,6]. Notably, among SAB-classified pregnancies in the Ailes cohort, only 81.3 percent had SAB as the sole outcome type within the pregnancy episode, compared with 97.0 percent of IAB-classified pregnancies [6]. This means that nearly 1 in 5 SAB identified (18.7%) could plausibly be a different pregnancy outcome entirely but was labeled as a SAB because those diagnoses came first in the hierarchy.

To accurately identify early pregnancy outcomes, such as SAB or IAB, claims data requires further triaging pregnancies when diagnosis, procedure or DRG codes suggest multiple different pregnancy outcomes. We hypothesized that we could reduce ambiguity among unknown pregnancy outcomes by incorporating additional codes for gestational age and medications. The objective of our study was to adapt and systematically test a modified hierarchical algorithm for identifying SAB in claims data.

## Materials & methods

### Study design and data source

We conducted a retrospective cohort study of individuals with a pregnancy-related insurance claim using the Merative MarketScan Research Commercial Database from January 1, 2018, through December 31, 2024 [10]. MarketScan contains deidentified, longitudinal claims data from commercially insured individuals in the U.S., representing a large convenience sample of employers and health plans. Available data include dates and location of service, enrollee demographic characteristics, inpatient and outpatient diagnosis codes, procedure codes, DRG codes, and outpatient medication prescription claims.

We used the Centers for Disease Control and Prevention and Centers for Medicare and Medicaid Services definitions for pregnancy outcomes. SAB was defined as the spontaneous expulsion of a pregnancy before 20 completed weeks of gestation. IAB was defined as the intentional termination of pregnancy. Stillbirth was defined as fetal death at or after 20 completed weeks of gestation and prior to delivery. All International Classification of Disease, Tenth Revision, Clinical Modification (ICD-10-CM) diagnosis codes, Current Procedural Terminology (CPT) and ICD-10 Procedure Coding System (ICD-10-PCS) procedure codes, and DRG codes used to define each outcome are provided in S1 Table.

### Pregnancy identification in administrative claims data

Identifying pregnancy outcomes in administrative claims data requires integrating multiple code types that each capture a distinct dimension of a pregnancy episode. ICD-10-CM diagnosis codes describe the clinical condition prompting a healthcare encounter (e.g., spontaneous abortion, ectopic pregnancy). Procedure codes, including CPT and Healthcare Common Procedure Coding System (HCPCS) codes, describe the specific clinical services rendered (e.g., dilation and curettage, aspiration of uterine contents). DRG codes are applied to inpatient stays and aggregate a patient’s diagnosis and procedures into a single payment category reflecting expected resource consumption. National Drug Code (NDC) or HCPCS codes for outpatient prescriptions can substantiate or adjudicate conflicting diagnosis codes, particularly for pregnancies managed medically with mifepristone or misoprostol.

Because each code type captures a different dimension of a pregnancy episode, algorithms that classify pregnancy outcomes must specify an explicit priority ordering, both across outcome types (SAB vs. IAB) and across code types (whether a DRG code or ICD-10 takes precedence when they conflict). This hierarchical structure is essential for resolving the substantial proportion of pregnancies in claims data that carry codes consistent with more than one outcome type.

### Base algorithm: Ailes et al. (2023)

We conducted a review of published algorithms for identifying pregnancy outcomes in administrative claims data to identify a suitable framework for adaptation [6,7]. Candidate algorithms were evaluated against four pre-specified criteria: (1) use of ICD-10-CM coding, (2) applicability to commercial insurance claims, (3) use of a hierarchical outcome classification structure, and (4) incorporation of multiple code types (diagnosis, procedure, and DRG). The algorithm developed by Ailes et al. (2023) best satisfied these criteria and served as our base framework [6].

The Ailes et al. algorithm uses a 16-level hierarchy to assign an initial pregnancy outcome from claims data. The hierarchy assigns the highest priority to diagnosis codes (levels 1–7), followed by DRG codes (levels 8–10), then procedure codes (levels 11–15), and finally livebirth DRG codes (level 16). Within each tier, outcomes are ordered as: multiple birth including live birth(s) and stillbirth(s) (diagnosis tier only), linkage to an infant record (live birth), stillbirth, live birth, spontaneous abortion, induced abortion, and ectopic pregnancy. An important feature of the original hierarchy is that DRG codes for live birth are assigned the lowest priority of all 16 levels, used only when no other pregnancy-related code is present. This was done because live birth DRG codes are non-specific and may co-occur with postpartum procedure codes in ways that obscure the delivery outcome.

Despite its methodological rigor, the Ailes algorithm has several limitations relevant to the studies of SAB and IAB in the ICD-10 era. First, a substantial proportion of pregnancies, particularly those with early gestational age codes and no definitive procedure or DRG code, remained unclassified (i.e., assigned an unknown outcome). Second, the algorithm did not incorporate outpatient prescription data for mifepristone or misoprostol, which are increasingly important for distinguishing medication-managed IAB from SAB following the expansion of medication abortion access, and which can affirmatively identify IAB that generate no outcome diagnosis code. Third, the place of DRG: Live births at the lowest hierarchy level meant that postpartum procedures, such as a D&C performed after a documented delivery, could override a live birth classification when no linked infant record was available, resulting in false SAB or IAB assignments. Finally, early pregnancies resolved without any clinical intervention generated were left as unclassified.

### Modified Ailes algorithm

We applied four pre-specified modifications to the Ailes framework, hereafter referred to as the modified Ailes algorithm. The overarching goals were to: (1) reduce the proportion of pregnancies with unclassified outcomes; (2) test the sensitivity of key hierarchy assumptions to SAB and IAB classification; and (3) improve the accurate discrimination between SAB and IAB in the context of changing medication abortion access following the *Dobbs v. Jackson Women’s Health Organization* decision (June 24, 2022) [11,12]. Each modification is described below.

#### Modification 1: Elevation of live birth DRG to prevent postpartum misclassification.

In the original Ailes et al. hierarchy, DRG: live birth is assigned the lowest priority (level 16). This means that a postpartum D&C or uterine aspiration procedure occurring after a documented delivery could take precedence over the live birth classification when no linked infant record was available, a common scenario when infants are enrolled under a separate insurance plan. In the modified algorithm, DRG: live birth is elevated priority to appear immediately after the diagnosis-code tier for live birth and stillbirth, before any SAB, IAB, or ectopic codes are evaluated. Additionally, pregnancies with gestational age codes indicating ≥20 weeks that were initially classified as SAB were recoded as stillbirths, consistent with established clinical and surveillance definitions.

#### Modification 2: Classification of early pregnancy loss managed expectantly.

Pregnancies with a gestational age of fewer than 56 days (8 complete weeks) that were followed by a cessation of all pregnancy-related claims for 60 or more consecutive days, despite continuous insurance enrollment, were classified as SAB. This pattern is clinically consistent with early pregnancy loss managed expectantly, without surgical intervention or medication treatment, in which the patient may seek no further pregnancy-related care [1]. Because such pregnancies generate few codes capturing the pregnancy resolution, they would otherwise remain unclassified under the original Ailes et al. framework. The 60-day threshold was chosen to distinguish true pregnancy resolution from lapses in prenatal care, while remaining within the physiologically plausible window for first-trimester loss. We note that some pregnancies in this category may represent IAB paid for out-of-pocket, for which no insurance claim would be generated.

#### Modification 3: Incorporation of prescription claims for medication abortion.

Outpatient prescription claims for mifepristone and misoprostol, identified using NDC and HCPCS codes, were incorporated to serve two distinct functions in outcome classification. First, they were used to adjudicate pregnancies with conflicting SAB and IAB diagnosis codes when no definitive surgical procedure code was present. When both SAB and IAB diagnosis codes co-occurred and a mifepristone and/or misoprostol claim was identified within a clinically relevant time window, the pregnancy was reclassified as IAB. In the absence of such a prescription claim, the pregnancy retained its SAB classification, consistent with the default assumption in the original Ailes et al. hierarchy that procedure codes used for IAB management are also used to treat SAB, whereas the converse is less common [5,6].

Second, mifepristone and misoprostol claims were used as affirmative evidence of IAB to classify pregnancies that the Ailes et al. algorithm would have left with an unknown outcome. These were pregnancies with no pregnancy outcome code, or only a gestational age dating code without any accompanying diagnosis, procedure, or DRG code indicating a specific outcome. In these cases, when a gestational age code indicated a pregnancy of fewer than 12 weeks and a mifepristone or misoprostol claim appeared within 7 days of the gestational age dating claim, the pregnancy was classified as IAB. This approach captures medication abortions that are managed entirely in the outpatient setting without a diagnosis code explicitly documenting the abortion, a pattern that may be increasingly common following the expansion of telehealth-based medication abortion access in the post-Dobbs period [12]

#### Modification 4: Expansion of the SAB diagnosis code set to include threated abortion and early pregnancy hemorrhage.

The Ailes et al. algorithm identifies SAB primarily through codes that explicitly denote pregnancy loss, including O02.1 (missed abortion) and the O03.x code series (spontaneous abortion). It does not incorporate diagnosis codes that describe the clinical presentation immediately preceding loss, specifically, hemorrhage in early pregnancy (O20.x). In our review of claims data, we identified a clinically important pattern in which a SAB-associated procedure code was billed alongside an O20.x diagnosis code rather than an O02.x or O03.x code. To capture these pregnancies, we expanded the SAB diagnosis code set to include O20.x and O26.85x codes under two conditions: (1) the O20.x or O26.85x code appeared in the absence of any other pregnancy outcome code (i.e., no co-occurring live birth, stillbirth, IAB, or ectopic diagnosis, procedure, or DRG code), and (2) no further pregnancy-related claims were present within the pregnancy episode window as defined by Ailes et al, indicating that the pregnancy did not continue to a later recorded outcome. When both conditions were met, the pregnancy episode was classified as SAB. When a SAB-specific procedure code co-occurred with an O20.x diagnosis code in the absence of other outcome codes, the procedural evidence was used as additional corroboration of the SAB classification. Pregnancies meeting the O20.x criteria that also carried any other outcome code were classified according to the standard hierarchy and were not affected by this modification.

### Testing hierarchy assumptions (Algorithms A, B, and C)

To evaluate the sensitivity of pregnancy outcome classification to the assumed priority of SAB over IAB within each code-type tier, we developed three additional algorithm variants (Algorithms A, B, and C). Each variant builds cumulatively on the prior, progressively inverting the SAB-IAB ordering across code-type tiers. These modifications apply exclusively to pregnancy episodes in which both SAB and IAB codes of the same type are present. Episodes with only one abortion code type are unaffected. All three variants incorporate the same four post-processing modifications as the modified Ailes algorithm.

Algorithm A inverts the diagnosis-code tier ordering, elevating IAB above SAB at levels 5–6 (from the original SAB → IAB to IAB → SAB). This modification theoretically increases the specificity of SAB ascertainment and the sensitivity of IAB ascertainment among pregnancies with conflicting diagnosis codes. Algorithm B additionally inverts the DRG-tier ordering, elevating DRG: IAB above DRG: SAB (levels 8–9), extending the same logic to inpatient stays with conflicting DRG codes. Algorithm C further inverts the procedure-code tier ordering, elevating procedure-coded IAB above procedure-coded SAB (levels 12–13), representing the most aggressive reclassification toward IAB and applying the inversion across all three code-type tiers simultaneous. A complete structured comparison of all five algorithms is provided in Table 1.

**Table 1:** Algorithms for identify pregnancy outcomes using MarketScan commercial claims data, January 1, 2018 – December 31, 2024 (all 50 states, Washington, D.C., and Puerto Rico).

Algorithm	Outcome Hierarchy (Code Type: Outcome)	Key Distinctions from Ailes Algorithm
Ailes et al. (2023)	1. Diagnosis: Multiple births including live birth(s) and stillbirths(s) 2. Linkage to infant record: Live birth 3. Diagnosis: Live birth 4. Diagnosis: Stillbirth 5. Diagnosis: Spontaneous abortion 6. Diagnosis: Induced abortion 7. Diagnosis: Ectopic pregnancy 8. DRG: Spontaneous abortion 9. DRG: Induced abortion 10. DRG: Ectopic pregnancy 11. Procedure: Stillbirth 12. Procedure: Spontaneous abortion 13. Procedure: Induced abortion 14. Procedure: Ectopic pregnancy 15. Procedure: Live birth 16. DRG: Live birth	Base/reference algorithm.
Modified Ailes	1–4. Same as Ailes **5. DRG: Live birth** 6. Diagnosis: Spontaneous abortion 7. Diagnosis: Induced abortion 8. Diagnosis: Ectopic pregnancy 9. DRG: Spontaneous abortion 10. DRG: Induced abortion 11. DRG: Ectopic Pregnancy 12. Procedure: Stillbirth 13. Procedure: Spontaneous abortion 14. Procedure: Induced abortion 15. Procedure: Ectopic pregnancy 16. Procedure: Live birth	**(1)** DRG: Livebirth elevated above SAB/IAB/Ectopic levels to prevent postpartum D&C from overriding live birth classification when infant linkage fails. **(2)** Pregnancies <56 days gestation with no subsequent obstetric claims (≥60-day gap, continuous enrollment) → classified as SAB. **(3)** Pregnancies with dual SAB + ectopic codes → classified as SAB unless supported by ectopic-specific procedure or methotrexate claim. **(4)** Mifepristone/misoprostol NDC and HCPCS claims used in two ways: (a) to reclassify conflicting SAB + IAB pregnancies as IAB when no surgical procedure code is present; and (b) to affirmatively classify as IAB pregnancies with unknown outcome or gestational age dating only, when a mifepristone/misoprostol claim appears with 7 days of a gestational age code indicating <14 weeks gestation. **(5)** SAB code set expanded to include hemorrhage in early pregnancy (O20.x) and spotting complicating pregnancy (O26.85x) when appearing without any other outcome code and with no subsequent pregnancy claims within the Ailes-defined episode window (≥42 days from the end of non-live birth outcomes); co-occuring SAB procedure codes (e.g., D&C, uterine aspiration) used as additional corroboration.
Algorithm A	1–5. Same as Modified Ailes **6. Diagnosis: Induced abortion ← swapped** **7. Diagnosis: Spontaneous abortion ← swapped** 8–16. Same as Modified Ailes	Diagnosis-level swap only: IAB elevated above SAB at diagnosis code tier (levels 6–7). Applies only when both SAB and IAB diagnosis codes are present in the same pregnancy episode. Theoretically increases specificity of SAB ascertainment and sensitivity of IAB ascertainment at the diagnosis code level.
Algorithm B	1–5. Same as Modified Ailes **6. Diagnosis: IAB ← swapped (as in Alg. A)** **7. Diagnosis: SAB ← swapped (as in Alg. A)** 8. Diagnosis: Ectopic pregnancy **9. DRG: Induced abortion ← swapped** **10. DRG: Spontaneous abortion ← swapped** 11–16. Same as Modified Ailes	DRG-level swap added: IAB elevated above SAB at DRG tier (levels 9–10). Applies only with both SAB and IAB DRG codes are present in the same pregnancy episode. Theoretically further increases specificity of SAB and sensitivity of IAB ascertainment by resolving DRG-level conflicts in favor of IAB.
Algorithm C	1–5. Same as Modified Ailes **6. Diagnosis: IAB ← swapped (as in Alg. A/B)** **7. Diagnosis: SAB ← swapped (as in Alg. A/B)** 8. Diagnosis: Ectopic pregnancy **9. DRG: Induced abortion ← swapped (as in Alg. B)** **10. DRG: Spontaneous abortion ← swapped (as in Alg. B)** 11. DRG: Ectopic Pregnancy 12. Procedure: Stillbirth **13. Procedure: Induced abortion ← swapped** **14. Procedure: Spontaneous abortion ← swapped** 15–16. Same as Modified Ailes	Procedure-level swap added: IAB elevated above SAB at procedure code tier (levels 13–14). Applies only when both SAB and IAB procedure codes (CPT or ICD-10-PCS) are presented in the same pregnancy episode. Represents the most aggressive reclassification toward IAB at all three code-type levels. Theoretically maximizes specificity of SAB and sensitivity of IAB across all code types.

### Statistical analysis

We first compared the distribution of pregnancy outcomes across all five algorithms to characterize how classification decisions affect outcome frequencies. We then conducted a series of sensitivity analysis to assess whether SAB and IAB rates differed by algorithm according to year, U.S. Census region, insurance type, site of diagnosis (outpatient vs. emergency department vs. inpatient), and the presence of NDC/HCPCS claims for mifepristone and misoprostol.

To evaluate temporal trends in SAB and IAB ascertainment surrounding the *Dobbs* decision, we calculated rates of SAB and IAB per 1,000 pregnancies by year across the full study period (January 1, 2018 – December 31, 2024). All datasets were constructed using SAS v9.4 (Cary, NC), and analyses were conducted in R (v4.5.1). This study was approved by the institutional review board at OHSU.

## Results

### Reducing proportion of unclassified pregnancy outcomes

The differences in defined pregnancy outcomes between the original Ailes algorithm and our Modified Ailes algorithm are shown in Table 2. The Modified Ailes algorithm reduced the proportion of unclassified pregnancy outcomes from 17.0% to 13.4%. The proportion of IAB identified by the Modified Ailes algorithm remained low at only 3.2% of pregnancies. Fig 1 shows how the Modified Ailes algorithm re-classified previously unclassified pregnancy outcomes into known pregnancy outcomes. The largest reduction in unclassified pregnancies came from re-classifying pregnancies as SAB. The Modified Algorithm re-classified 68,531 pregnancies as SAB that were previously unclassified (S2 Table). This was followed by an additional 21,178 pregnancies that were previously unclassified and able to be classified as live births by the Modified Algorithm. The Modified Algorithm also improved the specificity of the live birth outcome with 1,555 pregnancies formerly classified as SAB, re-classified as live birth. Only three pregnancies with an unclassified outcome were re-classified as IAB by the Modified Algorithm.

**Table 2:** Comparison of pregnancy outcome classification between the Ailes and modified Ailes algorithms using MarketScan commercial claims data, January 1, 2018 – December 31, 2024 (all 50 states, Washington, D.C., and Puerto Rico).

	Ailes^1^	Modified Ailes
**Individuals with known outcome date, N**	1,659,521	1,721,027
**Pregnancies with known outcome date, N**	2,008,211	2,093,776
**Live Births, N (%)**	1,594,062 (79.4%)	1,617,183 (77.2%)
**Still Births, N (%)**	12,288 (0.6%)	14,753 (0.7%)
**Live Birth + Still Birth, N (%)**	542 (0.03%)	542 (0.03%)
**Spontaneous Abortion, N (%)**	299,623 (14.9%)	366,599 (17.5%)
**Induced Abortion, N (%)**	68,242 (3.4%)	68,084 (3.2%)
**Ectopic Pregnancy, N (%)**	25,924 (1.3%)	25,754 (1.2%)
**Unclassified Pregnancy Outcomes (with known outcome date), N (%)** ^2^	7,530 (0.4%)	860 (0.04%)
**Unclassified Pregnancy Outcomes (with unknown outcome date), N (%)**	410,775 (17.0%)	325,207 (13.4%)
**Individuals Total, N**	1,963,900	1,963,900
**Pregnancies Total, N**	2,418,986	2,418,986

^1^Ailes EC, Zhu W, Clark EA, Huang Y-IA, Lampe MA, Kourtis AP, Et al. (2023) Identification of pregnancies and their outcomes in healthcare claims data, 2008–2019: An algorithm. PLoS ONE 18(4): e0284893.

^2^Unclassified outcomes include pregnancies with incomplete or conflicting codes that could not be definitively classified.

**Fig 1:**
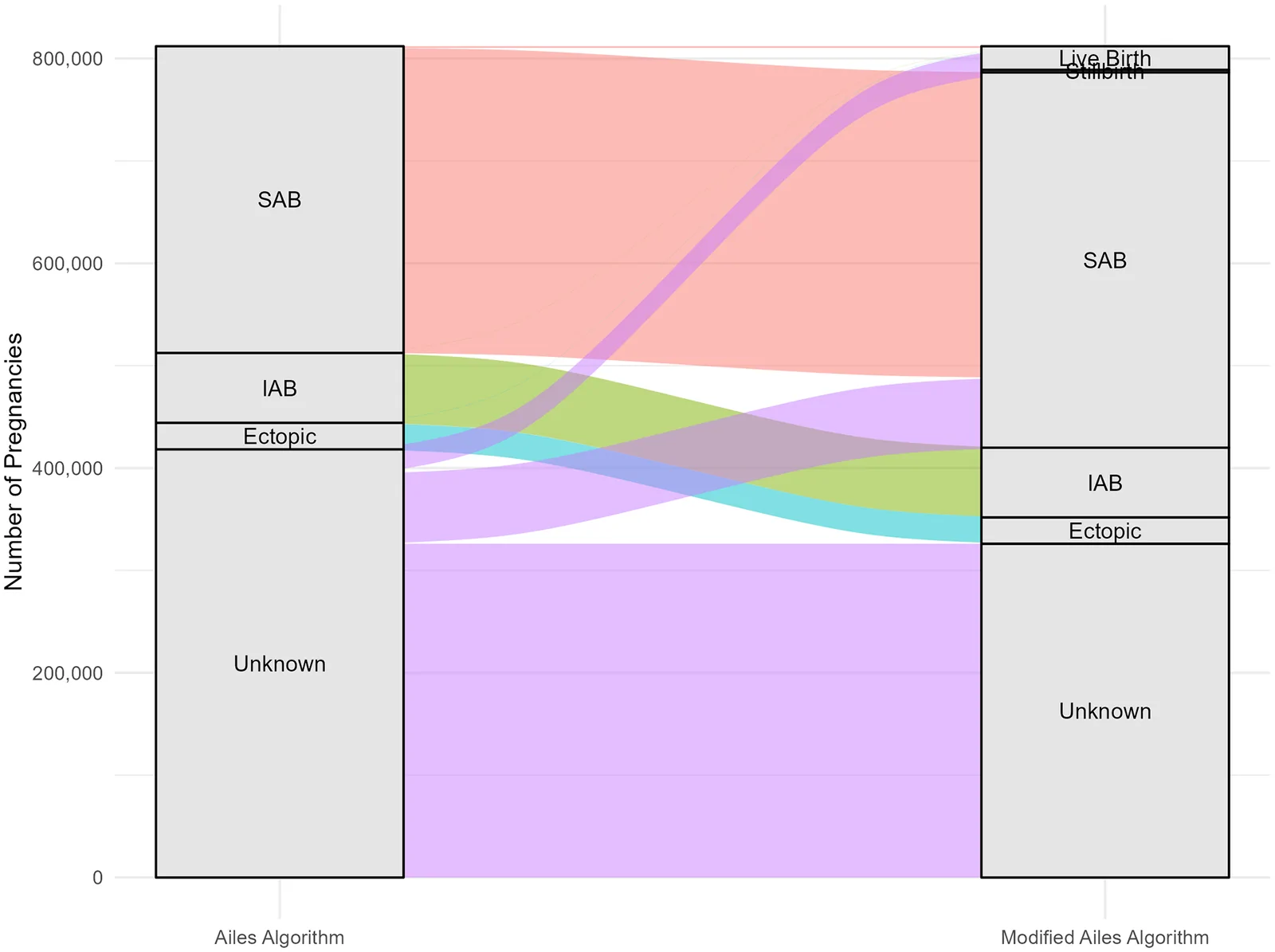
Reclassification of pregnancy outcomes, comparing Ailes and modified Ailes algorithms.

### Testing hierarchical assumptions (Algorithms A, B, and C)

Table 3 depicts the proportion of pregnancy outcomes after we made hierarchical adjustments to the Modified Algorithm (Algorithms A, B, and C). When we switched the diagnosis codes for SAB and IAB to prioritize IAB in the hierarchy, Algorithms A identified 8,645 pregnancies as IAB compared to SAB. We did not observe any additional changes when altering the order of the DRG codes for SAB and IAB and Algorithms A and B were equivalent. In Algorithm C, the procedure codes for SAB and IAB were swapped, and an additional 40 pregnancies were re-classified as IAB instead of SAB. The ICD codes used for SAB identification were more diverse than those used to identify IAB (S3 and S4 Tables). ICD code ‘Z332’ (encounter for elective termination of pregnancy) identified 94 percent of IAB with the remainder of IAB codes being used less than 1 percent of the time. Conversely, the most common ICD-10 codes for SAB were used between 10 and 45 percent of the time.

**Table 3:** Pregnancy outcome classification under progressive inversion of the spontaneous abortion-induced abortion hierarchy: comparison of Algorithms A, B, and C using MarketScan commercial claims data, January 1, 2018 – December 31, 2024 (all 50 states, Washington, D.C., and Puerto Rico).

	Algorithm A^1^	Algorithm B^2^	Algorithm C^3^
**Individuals with known outcome date, N**	1,721,027	1,721,027	1,721,027
**Pregnancies with known outcome date, N**	2,093,776	2,093,776	2,093,776
**Live Births, N (%)**	1,617,183 (77.2%)	1,617,183 (77.2%)	1,617,183 (77.2%)
**Still Births, N (%)**	14,665 (0.7%)	14,665 (0.7%)	14,664 (0.5%)
**Live Birth + Still Birth, N (%)**	542 (0.03%)	542 (0.03%)	542 (0.03%)
**Spontaneous Abortion, N (%)**	358,065 (17.1%)	358,065 (17.1%)	358,026 (17.1%)
**Induced Abortion, N (%)**	76,707 (3.7%)	76,707 (3.7%)	76,747 (3.7%)
**Ectopic Pregnancy, N (%)**	25,754 (1.2%)	25,754 (1.2%)	25,754 (1.2%)
**Unclassified Pregnancy Outcomes (with known outcome date), N (%)** ^4^	860 (0.04%)	860 (0.04%)	860 (0.04%)
**Unclassified Pregnancy Outcomes (with unknown outcome date), N (%)**	325,207 (13.4%)	325,207 (13.4%)	325,207 (13.4%)
**Individuals Total, N**	1,963,900	1,963,900	1,963,900
**Pregnancies Total, N**	2,418,986	2,418,986	2,418,986

^1^Pregnancies with both a spontaneous abortion (SAB) and induced abortion (IAB) diagnosis code are assigned as IAB.

^2^Pregnancies with both SAB and IAB diagnosis codes and procedure codes are assigned as IAB.

^3^Pregnancies with both SAB and IAB diagnosis codes, procedure codes, and DRGs are assigned as IAB.

^4^Unclassified outcomes include pregnancies with incomplete or conflicting codes that could not be definitively classified.

### Prevalence of SAB versus IAB outcome pre- and post-*Dobbs* decision

We did not observe significant differences in demographics or clinical characteristics between algorithm identification of SAB and IAB (S5 Table); however, we did see key consistent differences within the algorithms between SAB and IAB. A higher proportion of IAB compared to SAB was identified in the West and Northeast geographic regions (43% IAB versus 16–17% SAB) compared to the South (45–47% SAB versus 18–19% IAB). The majority of both SAB and IAB outcomes were identified in the outpatient setting. Across all algorithms, over 80 percent of IAB outcomes had codes for both mifepristone and misoprostol while roughly 90 percent of identified SAB only had misoprostol only codes. The rates of SAB and IAB remained consistent between algorithms but did change over time (S1 and S2 Figs). SAB rates per 1,000 pregnancies spiked starting in 2022 where IAB rates decreased in 2021 followed by a subsequent increase.

## Discussion

Administrative claims data are a critical tool for evaluating the downstream effects of abortion restrictions at the population level, but the validity of such analyses depends on the accuracy of the underlying algorithm used to classify pregnancy outcomes. Our analysis demonstrates that it is possible to adjust hierarchical algorithms to reduce the proportion of unclassified pregnancy outcomes. Our modified algorithm reduced the proportion of unclassified pregnancy outcomes by four percent and increased the proportion of SAB to 17 percent. This is an improvement compared to other methods but may still underestimate the true incidence of SAB [2,13]. We identified more SAB that were previously unclassified by identifying early miscarriages that had been managed expectantly and classifying them as SAB, incorporating medication codes for mifepristone and misoprostol, and expanding the SAB diagnosis codes to include clinical scenarios that lead to SAB such as threatened abortion and early pregnancy bleeding. By moving the DRG for live birth higher up in the hierarchy we improved the specificity for the live birth outcome that was previously being conflated as SAB or IAB. This is consistent with previous evidence that shows that DRG codes may be more useful in correctly classifying more complicated pregnancies that are more likely to contain conflicting ICD codes [14].

Identifying IAB in claims data remains challenging [3,15,16]. All our algorithms identified less than five percent of pregnancies as IAB which is almost certainly a gross underestimate of the true IAB incidence [17]. This is likely due to IAB occurring outside the billing system as insurance coverage for IAB is limited even among private payors such as in our dataset [3]. Researchers must be cautious of using claims data to make assumptions about IAB outcomes. We saw higher rates of IAB in the Northeast and West U.S. regions compared to the Southern regions which may suggest that IAB identification in claims data is more useful in states without significant restrictions to abortion care.

Less than one percent of pregnancy outcomes had dual codes for both IAB and SAB so our hierarchical adjustments only modestly increased the proportion of IAB outcomes identified. Outcomes with duplicate IAB and SAB codes may be a result of a coding error or represent a complex clinical scenario that ultimately resulted either in a decision to terminate or a pregnancy loss such as pre-viable rupture of membranes or pre-viable placental abruption. The SAB ICD-10 codes represent a variety of clinical scenarios that may lead to pregnancy loss. However, the IAB ICD-10 codes represent the potential complications from an abortion, but not the reason someone may pursue abortion care (S2 and S3 Tables). Outside of the ICD-10 codes for pregnancy ambivalence (Z64.0) and fetal anomalies (O35.X), no such codes exist. While there are ICD-10 codes for social determinants of health (poverty, food insecurity, housing instability), that may lead someone to seek abortion care, these are non-specific and their inclusion would be reductive of the broad and often nuanced reasons an individual pursues abortion [18].

Although the rate of IAB and SAB identified over time remained consistent across algorithms, we noted two unanticipated temporal shifts. In 2021, there was a decrease in the number of IAB identified which may represent a COVID-19 shift to alternative delivery for abortion care such as telemedicine access which is likely to be under-captured in insurance claims [19]. The identified SAB rate also increased from 2022 to 2024. Vital statistics data beyond 2019 has not been published, so it is unknown if this is a true biologic trend [20]. Instead, the increase may reflect a post-*Dobbs* trend of patients seeking SAB care within healthcare settings that more commonly bill insurance due to access issues, or IAB being miscoded as SAB to avoid legal persecution. However, we did see a reciprocal decrease in IAB claims.

Strengths of this study include the use of ICD-10 codes which have not previously studied without the inclusion of the less specific and older ICD-9 codes. We also used a large multi-year national sample through Marketscan. Limitations include a lack of adjudication to determine the true sensitivity and specificity for identifying SAB versus IAB outcomes in our dataset. Instead, we relied on the high levels of internal consistency between algorithms [9]. Our algorithms also do not identify exact gestational age which remains difficult in claims for outcomes that occur early in pregnancy [21]. Methodological efforts like the #WeCount report rely on clinical provider data to bypass claims-based ambiguity, but they lack the longitudinal patient-level detail found in claims data that allows for more robust analyses [22].

## Conclusion

In the post-*Dobbs* era, it is critical to identify early pregnancy outcomes accurately and with transparency. Understanding the strengths and limitations of the various methodologies to identify SAB and IAB will improve the integrity of claims-based research for early pregnancy outcomes.

## Supporting information

S1 Table Pregnancy identification codes. (XLSX)

S2 Table Comparison of pregnancy outcome reclassification between Ailes and modified Ailes algorithms. (DOCX)

S3 Table Frequency of diagnosis codes among people with a diagnosis of spontaneous abortion. (DOCX)

S4 Table Frequency of diagnosis codes among pregnancies with a diagnosis of induced abortion. (DOCX)

S5 Table Demographics of SAB vs. IAB outcome by algorithm, January 1, 2018 – December 31, 2024 (all 50 states, Washington, D.C., and Puerto Rico). (DOCX)

S1 Fig Spontaneous abortion (SAB) rates per 1,000 pregnancies, 2018–2024, all US States, Washington D.C. (TIF)

S2 Fig Induced abortion (IAB) rates per 1,000 pregnancies, 2018–2024, all US States, Washington D.C. (TIF)
