# Supplementary material for: Sensitivity of spontaneous abortion classification to hierarchical algorithm assumptions in US commercial claims data, 2018–2024
Source: PLoS One. 2026 Oct 28;21(10):e0361330. doi: 10.1371/journal.pone.0361330 (PMC13716177; doi:10.1371/journal.pone.0361330)
Supplement: Supplementary file 1 [file pone.0361330.s002.docx]

**S2 Table: Comparison of Pregnancy Outcome Reclassification between Ailes and Modified Ailes Algorithms**

| **Ailes Algorithm** | **Modified Ailes Algorithm** | **N** |
| --- | --- | --- |
| Live Birth | Live Birth | 1,594,062 |
| Live Birth + Stillbirth | Live Birth + Stillbirth | 542 |
| Stillbirth | Stillbirth | 12,288 |
| SAB | Live Birth | 1,555 |
| SAB | SAB | 298,068 |
| IAB | Live Birth | 161 |
| IAB | IAB | 68,081 |
| Ectopic | Live Birth | 172 |
| Ectopic | Ectopic | 25,752 |
| Unknown | Live Birth | 21,178 |
| Unknown | Still Birth | 2,518 |
| Unknown | SAB | 68,531 |
| Unknown | IAB | 3 |
| Unknown | Ectopic | 2 |
| Unknown | Unknown | 326,045 |
