# Supplementary material for: Sensitivity of spontaneous abortion classification to hierarchical algorithm assumptions in US commercial claims data, 2018–2024
Source: PLoS One. 2026 Oct 28;21(10):e0361330. doi: 10.1371/journal.pone.0361330 (PMC13716177; doi:10.1371/journal.pone.0361330)
Supplement: Supplementary file 2 [file pone.0361330.s003.docx]

**S3 Table: Frequency of Diagnosis Codes Among People with a Diagnosis of Spontaneous Abortion**

| **Code** | **Description** | **N** | **%** |
| --- | --- | --- | --- |
| O02 | Other abnormal products of conception | 134 | 0.04% |
| O020 | Blighted ovum and nonhydatidiform mole | 12199 | 3.30% |
| O021 | Missed abortion | 175992 | 47.57% |
| O028 | Other specified abnormal products of conception | 2 | 0.00% |
| O0281 | Inappropriate change in quantitative hCG in early pregnancy | 6854 | 1.85% |
| O0289 | Other abnormal products of conception | 3725 | 1.01% |
| O029 | Abnormal product of conception, unspecified | 4039 | 1.09% |
| O03 | Spontaneous abortion | 99 | 0.03% |
| O030 | Genital tract and pelvic infection following incomplete spontaneous abortion | 673 | 0.18% |
| O031 | Delayed or excessive hemorrhage following incomplete spontaneous abortion | 5045 | 1.36% |
| O032 | Embolism following incomplete spontaneous abortion | 292 | 0.08% |
| O0330 | Unspecified complication following incomplete spontaneous abortion | 814 | 0.22% |
| O0331 | Shock following incomplete spontaneous abortion | 141 | 0.04% |
| O0332 | Rental failure following incomplete spontaneous abortion | 5 | 0.00% |
| O0333 | Metabolic disorder following incomplete spontaneous abortion | 159 | 0.04% |
| O0334 | Damage to pelvic organs following incomplete spontaneous abortion | 33 | 0.01% |
| O0335 | Other venous complications following incomplete spontaneous abortion | 14 | 0.00% |
| O0336 | Cardiac arrest following incomplete spontaneous abortion | 6 | 0.00% |
| O0337 | Sepsis following incomplete spontaneous abortion | 450 | 0.12% |
| O0338 | Urinary tract infection following incomplete spontaneous abortion | 275 | 0.07% |
| O0339 | Incomplete spontaneous abortion with other complications | 2043 | 0.55% |
| O034 | Incomplete spontaneous abortion without complication | 66034 | 17.85% |
