# Supplementary material for: Sensitivity of spontaneous abortion classification to hierarchical algorithm assumptions in US commercial claims data, 2018–2024
Source: PLoS One. 2026 Oct 28;21(10):e0361330. doi: 10.1371/journal.pone.0361330 (PMC13716177; doi:10.1371/journal.pone.0361330)
Supplement: Supplementary file 3 [file pone.0361330.s004.docx]

**S4 Table: Frequency of Diagnosis Codes Among Pregnancies with a Diagnosis of Induced Abortion**

| **Code** | **Description** | **N** | **%** |
| --- | --- | --- | --- |
| O045 | Genital tract and pelvic infection following (induced) termination of pregnancy | 70 | 0.10% |
| O046 | Delayed or excessive hemorrhage following (induced) termination of pregnancy | 368 | 0.54% |
| O047 | Embolism following (induced) termination of pregnancy | 6 | 0.01% |
| O0480 | (Induced) termination of pregnancy with unspecified complications | 556 | 0.82% |
| O0481 | Shock following (induced) termination of pregnancy | 3 | 0.00% |
| O0482 | Renal failure following (induced) termination of pregnancy | 7 | 0.01% |
| O0483 | Metabolic disorder following (induced) termination of pregnancy | 11 | 0.02% |
| O0484 | Damage to pelvic organs following (induced) termination of pregnancy | 24 | 0.04% |
| O0485 | Other venous complications following (induced) termination of pregnancy | 3 | 0.00% |
| O0486 | Cardiac arrest following (induced) termination of pregnancy | 2 | 0.00% |
| O0487 | Sepsis following (induced) termination of pregnancy | 12 | 0.02% |
| O0488 | Urinary tract infection following (induced) termination of pregnancy | 29 | 0.04% |
| O0489 | (Induced) termination of pregnancy with other complications | 382 | 0.56% |
| O070 | Genital tract and pelvic infection following failed attempted termination of pregnancy | 30 | 0.04% |
| O071 | Delayed or excessive hemorrhage following failed attempted termination of pregnancy | 193 | 0.28% |
| O0730 | Failed attempted termination of pregnancy with unspecified complications | 17 | 0.02% |
| O0731 | Shock following failed attempted termination of pregnancy | 2 | 0.00% |
| O0732 | Renal failure following failed attempted termination of pregnancy | 3 | 0.00% |
| O0733 | Metabolic disorder following failed attempted termination of pregnancy | 1 | 0.00% |
| O0734 | Damage to pelvic organs following failed attempted termination of pregnancy | 1 | 0.00% |
| O0735 | Other venous complications following failed attempted termination of pregnancy | 0 | 0.00% |
| O0736 | Cardiac arrest following failed attempted termination of pregnancy | 0 | 0.00% |
| O0737 | Sepsis following failed attempted termination of pregnancy | 4 | 0.01% |
| O0738 | Urinary tract infection following failed attempted termination of pregnancy | 7 | 0.01% |
| O0739 | Failed attempted termination of pregnancy with other complications | 40 | 0.06% |
| O074 | Failed attempted termination of pregnancy without complication | 527 | 0.77% |
| Z332 | Encounter for elective termination of pregnancy | 64621 | 94.91% |
