# Supplementary material for: Sensitivity of spontaneous abortion classification to hierarchical algorithm assumptions in US commercial claims data, 2018–2024
Source: PLoS One. 2026 Oct 28;21(10):e0361330. doi: 10.1371/journal.pone.0361330 (PMC13716177; doi:10.1371/journal.pone.0361330)
Supplement: Supplementary file 4 [file pone.0361330.s005.docx]

**S5 Table: Demographics of SAB vs. IAB Outcome by Algorithm, January 1, 2018 – December 31, 2024 (All 50 states, Washington, D.C., and Puerto Rico)**

|  | **Ailes** | | **Modified Ailes** | | **Algorithm A** | | **Algorithm B** | | **Algorithm C** | |
| --- | --- | --- | --- | --- | --- | --- | --- | --- | --- | --- |
| **Characteristic** | **SAB**  N = 299,623 | **IAB**  N = 68,242 | **SAB**  N = 366,599 | **IAB**  N = 68,084 | **SAB**  N = 358,065 | **IAB**  N = 76,707 | **SAB**  N = 358,065 | **IAB**  N = 76,707 | **SAB**  N = 358,026 | **IAB**  N = 76,747 |
| **Age, mean (SD)** | 32.24 (6.08) | 29.31 (6.62) | 31.73 (6.18) | 29.31 (6.62) | 31.76 (6.16) | 29.48 (6.64) | 31.76 (6.16) | 29.48 (6.64) | 31.75 (6.16) | 29.48 (6.64) |
| **Age (years)** |  |  |  |  |  |  |  |  |  |  |
| 15-19 | 5,224 (1.7%) | 3,364 (4.9%) | 7,726 (2.1%) | 3,358 (4.9%) | 7,409 (2.1%) | 3,677 (4.8%) | 7,409 (2.1%) | 3,677 (4.8%) | 7,409 (2.1%) | 3,677 (4.8%) |
| 20-24 | 30,471 (10.2%) | 15,880 (23.3%) | 43,542 (11.9%) | 15,818 (23.2%) | 42,030 (11.7%) | 17,340 (22.6%) | 42,030 (11.7%) | 17,340 (22.6%) | 42,026 (11.7%) | 17,345 (22.6%) |
| 25-29 | 60,347 (20.2%) | 16,802 (24.6%) | 79,053 (21.6%) | 16,763 (24.6%) | 77,253 (21.6%) | 18,577 (24.3%) | 77,253 (21.6%) | 18,577 (24.3%) | 77,251 (21.6%) | 18,579 (24.2%) |
| 30-34 | 92,179 (30.8%) | 15,598 (22.9%) | 110,517 (30.2%) | 15,562 (22.9%) | 108,326 (30.3%) | 17,782 (23.2%) | 108,326 (30.3%) | 17,782 (23.2%) | 108,316 (30.3%) | 17,792 (23.2%) |
| 35-39 | 75,352 (25.1%) | 11,507 (16.9%) | 85,675 (23.3%) | 11,497 (16.9%) | 83,822 (23.4%) | 13,376 (17.5%) | 83,822 (23.4%) | 13,376 (17.5%) | 83,806 (23.4%) | 13,392 (17.5%) |
| 40-44 | 32,015 (10.7%) | 4,528 (6.6%) | 35,431 (9.6%) | 4,523 (6.6%) | 34,666 (9.7%) | 5,296 (6.9%) | 34,666 (9.7%) | 5,296 (6.9%) | 34,660 (9.7%) | 5,302 (6.9%) |
| 45-49 | 3,807 (1.3%) | 452 (0.6%) | 4,347 (1.2%) | 452 (0.7%) | 4,260 (1.2%) | 539 (0.7%) | 4,260 (1.2%) | 539 (0.7%) | 4,259 (1.2%) | 540 (0.7%) |
| Unknown | 228 (0.1%) | 111 (0.2%) | 308 (0.1%) | 111(0.2%) | 299 (0.1%) | 120 (0.2%) | 299 (0.1%) | 120 (0.2%) | 299 (0.1%) | 120 (0.2%) |
| **Encounter Year** |  |  |  |  |  |  |  |  |  |  |
| 2018 | 49,126 (16.4%) | 11,134 (16.3%) | 58,493 (16.0%) | 11,120 (16.3%) | 57,252 (16.0%) | 12,375 (16.1%) | 57,252 (16.0%) | 12,375 (16.1%) | 57,245 (16.0%) | 12,382 (16.1%) |
| 2019 | 44,730 (14.9%) | 10,652 (15.6%) | 53,576 (14.6%) | 10,623 (15.6%) | 52,305 (14.6%) | 11,902 (15.5%) | 52,305 (14.6%) | 11,902 (15.5%) | 52,297 (14.6%) | 11,910 (15.5%) |
| 2020 | 40,669 (13.6%) | 9,200 (13.5%) | 48,075 (13.1%) | 9,174 (13.5%) | 47,005 (13.1%) | 10,255 (13.4%) | 47,005 (13.1%) | 10,255 (13.4%) | 47,003 (13.1%) | 10,257 (13.4%) |
| 2021 | 40,373 (13.5%) | 8,400 (12.3%) | 48,522 (13.2%) | 8,379 (12.3%) | 47,565 (13.3%) | 9,346 (12.2%) | 47,565 (13.3%) | 9,346 (12.2%) | 47,561 (13.3%) | 9,350 (12.2%) |
| 2022 | 37,911 (12.7%) | 8,693 (12.7%) | 45,549 (12.4%) | 8,668 (12.7%) | 44,434 (12.4%) | 9,800 (12.8%) | 44,434 (12.4%) | 9,800 (12.8%) | 44,430 (12.4%) | 9,804 (12.8%) |
| 2023 | 41,732 (13.9%) | 9,636 (14.1%) | 50,814 (13.9%) | 9,613 (14.1%) | 49,349 (13.8%) | 11,090 (14.5%) | 49,349 (13.8%) | 11,090 (14.5%) | 49,340 (13.8%) | 11,100 (14.5%) |
| 2024 | 45,082 (15.0%) | 10,527 (15.4%) | 61,570 (16.8%) | 10,507 (15.4%) | 60,155 (16.8%) | 11,939 (15.6%) | 60,155 (16.8%) | 11,939 (15.6%) | 60,150 (16.8%) | 11,944 (15.6%) |
| **US Geographic Region** |  |  |  |  |  |  |  |  |  |  |
| Northeast | 53,103 (17.7%) | 29,758 (43.7%) | 62,014 (16.9%) | 29,704 (43.7%) | 58,603 (16.4%) | 33,141 (43.3%) | 58,603 (16.4%) | 33,141 (43.3%) | 58,593 (16.4%) | 33,152 (43.3%) |
| Midwest | 55,797 (18.6%) | 7,879 (11.6%) | 68,622 (18.7%) | 7,850 (11.5%) | 67,404 (18.8%) | 9,088 (11.9%) | 67,404 (18.8%) | 9,088 (11.9%) | 67,400 (18.8%) | 9,092 (11.9%) |
| South | 137,514 (45.9%) | 12,565 (18.4%) | 171,879 (46.9%) | 12,535 (18.4%) | 169,806 (47.5%) | 14,638 (19.1%) | 169,806 (47.5%) | 14,638 (19.1%) | 169,791 (47.5%) | 14,653 (19.1%) |
| West | 52,280 (17.5%) | 17,828 (26.2%) | 62,968 (17.2%) | 17,784 (26.2%) | 61,171 (17.1%) | 19,594 (25.6%) | 61,171 (17.1%) | 19,594 (25.6%) | 61,161 (17.1%) | 19,604 (25.6%) |
| Unknown | 729 (0.2%) | 212 (0.1%) | 1,116 (0.2%) | 211 (0.1%) | 1,081 (0.2%) | 246 (0.2%) | 1,081 (0.2%) | 246 (0.2%) | 1,081 (0.2%) | 246 (0.2%) |
| **Rurality** |  |  |  |  |  |  |  |  |  |  |
| Urban | 230,418 (76.9%) | 59,391 (87.0%) | 282,771 (77.1%) | 59,816 (87.8%) | 273,354 (76.3%) | 66,520 (86.7%) | 273,354 (76.3%) | 66,520 (86.7%) | 273,322 (76.3%) | 66,553 (86.7%) |
| Rural | 25,764 (8.6%) | 2,122 (3.1%) | 32,716 (8.9%) | 2,112 (3.1%) | 32,386 (9.0%) | 2,447 (3.2%) | 32,386 (9.0%) | 2,447 (3.2%) | 32,382 (9.0%) | 2,451 (3.2%) |
| Unknown | 43,441 (14.5%) | 6,729 (5.5%) | 51,112 (14.0%) | 6,156 (9.1%) | 52,325 (14.7%) | 7,740 (10.1%) | 52,325 (14.7%) | 7,740 (10.1%) | 52,322 (14.7%) | 7,743 (10.1%) |
| **Insurance Type** |  |  |  |  |  |  |  |  |  |  |
| Managed Care | 81,344 (27.1%) | 26,908 (39.4%) | 100,953 (27.5%) | 26,853 (39.4%) | 97,820 (27.3%) | 30,009 (39.1%) | 97,820 (27.3%) | 30,009 (39.1%) | 97,807 (27.3%) | 30,022 (39.1%) |
| Provider Preferred | 135,292 (45.2%) | 26,493 (38.8%) | 165,108 (45.0%) | 26,433 (38.8%) | 161,571 (45.1%) | 30,008 (39.1%) | 161,571 (45.1%) | 30,008 (39.1%) | 161,554 (45.1%) | 30,026 (39.1%) |
| High Deductible | 71,154 (23.7%) | 12,589 (18.4%) | 85,467 (23.3%) | 12,553 (18.4%) | 83,881 (23.4%) | 14,160 (18.5%) | 83,881 (23.4%) | 14,160 (18.5%) | 83,872 (23.4%) | 14,169 (18.5%) |
| Other/Unknown | 11,833 (3.9%) | 2,252 (3.3%) | 15,071 (4.1%) | 2,245 (3.3%) | 14,793 (4.1%) | 2,530 (3.3%) | 14,793 (4.1%) | 2,530 (3.3%) | 14,793 (4.1%) | 2,530 (3.3%) |
| **Insurance Coverage** |  |  |  |  |  |  |  |  |  |  |
| Primary | 173,168 (57.8%) | 40,982 (60.2%) | 212,924 (58.1%) | 40,901 (60.2%) | 207,706 (58.1%) | 46,175 (60.3%) | 207,706 (58.1%) | 46,175 (60.3%) | 207,688 (58.1%) | 46,194 (60.3%) |
| Spouse | 98,738 (33.0%) | 9,585 (14.1%) | 114,632 (31.3%) | 9,572 (14.1%) | 112,925 (31.6%) | 11,303 (14.8%) | 112,925 (31.6%) | 11,303 (14.8%) | 112,908 (31.6%) | 11,320 (14.8%) |
| Dependent | 27,489 (9.2%) | 17,564 (25.8%) | 38,735 (10.6%) | 17,500 (25.7%) | 37,135 (10.4%) | 19,109 (25.0%) | 37,135 (10.4%) | 19,109 (25.0%) | 37,131 (10.4%) | 19,113 (24.9%) |
| Unknown | 228 (0.0%) | 111 (0.0%) | 308 (0.0%) | 111 (0.0%) | 299 (0.0%) | 120 (0.0%) | 299 (0.0%) | 120 (0.0%) | 299 (0.0%) | 120 (0.0%) |
| **Location of Diagnosis** |  |  |  |  |  |  |  |  |  |  |
| Outpatient | 232,996 (77.8%) | 63,355 (92.8%) | 271,227 (74.0%) | 63,228 (92.9%) | 264,953 (74.0%) | 70,587 (92.0%) | 264,953 (74.0%) | 70,587 (92.0%) | 264,937 (74.0%) | 70,599 (92.0%) |
| Emergency Department | 40,467 (13.5%) | 402 (0.6%) | 59,852 (16.3%) | 399 (0.6%) | 58,600 (16.4%) | 874 (1.1%) | 58,600 (16.4%) | 874 (1.1%) | 58,599 (16.4%) | 874 (1.1%) |
| Other | 25,021 (8.3%) | 3,281 (4.8%) | 25,823 (7.0%) | 3,252 (4.8%) | 24,820 (6.9%) | 4,038 (5.3%) | 24,820 (6.9%) | 4,038 (5.3%) | 24,815 (6.9%) | 4,040 (5.3%) |
| Unknown | 1,139 (0.4%) | 1,204 (1.8%) | 9,697 (2.7%) | 1,205 (1.7%) | 9,692 (2.7%) | 1,208 (1.6%) | 9,692 (2.7%) | 1,208 (1.6%) | 9,675 (2.7%) | 1,234 (1.6%) |
| **Medical Management** |  |  |  |  |  |  |  |  |  |  |
| Mifepristone plus Misoprostol | 4,807 (10.5%) | 27,009 (83.0%) | 4,814 (10.5%) | 26,972 (83.0%) | 2,670 (6.3%) | 29,125 (81.0%) | 2,670 (6.3%) | 29,125 (81.0%) | 2,665 (6.3%) | 29,130 (81.0%) |
| Misoprostol only | 40,919 (89.5%) | 5,535 (17.0%) | 41,232 (89.5%) | 5,526 (17.0%) | 39,953 (93.7%) | 6,823 (19.0%) | 39,953 (93.7%) | 6,823 (19.0%) | 39,950 (93.7%) | 6,826 (19.0%) |
| ^1^Mean (SD); n (%) | | | | | | | | | | |
