# Supplementary material for: Sensitivity of spontaneous abortion classification to hierarchical algorithm assumptions in US commercial claims data, 2018–2024
Source: PLoS One. 2026 Oct 28;21(10):e0361330. doi: 10.1371/journal.pone.0361330 (PMC13716177; doi:10.1371/journal.pone.0361330)
Supplement: Supplementary file 5 [file pone.0361330.s009.docx]

**Comments to the Author**

1. Is the manuscript technically sound, and do the data support the conclusions?

The manuscript must describe a technically sound piece of scientific research with data that supports the conclusions. Experiments must have been conducted rigorously, with appropriate controls, replication, and sample sizes. The conclusions must be drawn appropriately based on the data presented.

Reviewer #1: Yes

Reviewer #2: Yes

2. Has the statistical analysis been performed appropriately and rigorously?

Reviewer #1: Yes

Reviewer #2: Yes

3. Have the authors made all data underlying the findings in their manuscript fully available?

The [PLOS Data policy](https://urldefense.com/v3/__http://track.editorialmanager.com/CL0/http:*2F*2Fwww.plosone.org*2Fstatic*2Fpolicies.action*23sharing/1/010f019f07a7cbb4-7ab5f1d1-2e87-4235-bd22-4ca9891ed153-000000/Z7_a74YhB6sGXDDpJ5CCLTbMM2427Ov08NuraxkMYKM=258__;JSUlJSU!!Mi0JBg!LDNmqsiqG-DxL4srukvoZHbsr7-E-J1BxClhXs4Q2L_A44T2f3aMg69J1XY6-Kh7OtbRd8hGCQYQZpe6$) requires authors to make all data underlying the findings described in their manuscript fully available without restriction, with rare exception (please refer to the Data Availability Statement in the manuscript PDF file). The data should be provided as part of the manuscript or its supporting information, or deposited to a public repository. For example, in addition to summary statistics, the data points behind means, medians and variance measures should be available. If there are restrictions on publicly sharing data—e.g. participant privacy or use of data from a third party—those must be specified.

Reviewer #1: No

Reviewer #2: Yes

4. Is the manuscript presented in an intelligible fashion and written in standard English?

PLOS ONE does not copyedit accepted manuscripts, so the language in submitted articles must be clear, correct, and unambiguous. Any typographical or grammatical errors should be corrected at revision, so please note any specific errors here.

Reviewer #1: Yes

Reviewer #2: Yes

5. Review Comments to the Author

Please use the space provided to explain your answers to the questions above. You may also include additional comments for the author, including concerns about dual publication, research ethics, or publication ethics. (Please upload your review as an attachment if it exceeds 20,000 characters)

Reviewer #1: Overall, this is an easy-to-follow and much-needed evaluation of the Ailes algorithm and methods to improve specificity for early pregnancy outcomes, an area that has long represented a challenge in reproductive health research.

The most meaningful contribution of this work is the improved sensitivity for SAB achieved through the classification of previously unclassified pregnancy outcomes. The changes in discrimination between IAB and SAB are comparatively modest, but remain valuable to explore given the importance of accurately distinguishing these outcomes in reproductive health research. My suggestions are primarily minor, with the exception of the comment regarding the conservative episode definition and LMP assignment within the Ailes algorithm.

The manuscript is largely technically sound. I do have one additional question that I hope the authors may be able to address.

The authors appropriately focus on incorporating additional nonspecific codes for SAB into the algorithm, given the well-documented limitations of claims data for identifying SAB. However, the manuscript does not explore whether pregnancies with multiple outcomes occurring in close temporal proximity were observed. This may represent another source of missed ascertainment for both SAB and IAB. The Ailes algorithm is intentionally conservative in defining distinct pregnancy episodes (e.g., dif0, dif1, and dif2), and it is therefore unclear whether modifications to the livebirth DRG hierarchy would meaningfully affect SAB and IAB ascertainment beyond the reclassification of the 1,555 pregnancies discussed on line 275. Additional discussion of this issue would help readers better understand the extent to which residual misclassification may be driven by episode construction rather than outcome hierarchy.
**Response**: We thank the reviewer for this important observation. To identify records likely belonging to the same pregnancy, we followed the Ailes et al. approach and required ≥120 days from any live birth record and ≥42 days from the end of all other outcomes to the service date and inferred LMP of the subsequent pregnancy. We agree with the reviewer that we did not formally test the sensitivity of SAB and IAB ascertainment to these episode-construction thresholds, and that our hierarchy modifications operate entirely within the episode boundaries these thresholds define. Because our changes can only reclassify outcomes within episodes already established by these conservative gap windows, they cannot address misclassification arising from episode construction itself. We agree this may help explain why our live birth DRG modification produced a comparatively modest reclassification, and that the residual misclassification of both SAB and IAB may be attributable to episode segmentation rather than outcomes hierarchy. We have added a limitation to acknowledging this.

Lines 355-365: Our modifications operate entirely within the pregnancy episode boundaries defined by the original Ailes et al. algorithm, which required ≥120 days from any live birth or stillbirth record, and ≥42 days from any other pregnancy outcome, to the inferred LMP of the subsequent pregnancy. We did not test the sensitivity of SAB and IAB ascertainment to these thresholds or assess how often closely-spaced claims were split into separate episodes or merged into one. Thus, our hierarchy modifications can only reclassify outcomes within episodes already defined by these gaps and cannot recover misclassification introduced by episode constriction itself. The modest reclassification from elevating the live birth DRG (n = 1,555) may partly reflect this ceiling, with residual SAB/IAB misclassification driven by episode segmentation rather than outcome hierarchy. Future work should test sensitivity to alternative episode-window definitions to disentangle these two sources of error.

Specific questions/comments:
Line 64: Some additional specificity may be helpful here. While the policy implications are certainly important, the primary contribution of the present work appears to be improved ascertainment of previously unclassified outcomes rather than extensive reclassification between SAB and IAB. The statement may be strengthened by more explicitly describing how misclassification can affect epidemiologic estimates and interpretation of reproductive health trends. For example: Misclassification of spontaneous and induced abortion outcomes may distort epidemiologic evidence by underestimating their occurrence and obscuring distinctions between them. Consequently, observed trends over time and evaluations of reproductive health policies may be biased, limiting the ability to accurately assess patterns of spontaneous and iatrogenic abortion.

**Response**: We agree with this adjustment, and this has been re-worded.

Lines 63-67: Misclassification of SAB and IAB outcomes may distort epidemiologic evidence by underestimating their occurrence and obscuring distinctions between them. Consequently, observed trends over time and evaluations of reproductive health policies may be biased, limiting the ability to accurately assess patterns of spontaneous and iatrogenic abortion.

Line 75: Reference #149 has a formatting error

**Response:** Thank you for catching this error. This has been updated.

Line 86-87 are the most compelling justifications for adjusting the SAB IAB hierarchy
Line 156: Can the authors please explain a bit more detail how/why outpatient mifepristone and misoprostol can affirmatively identify IAB if there is no outcome code? Do the authors mean mife and miso outpatient prescriptions associated with a Z332 but no outcome code? If so I would state this, there are cases where a loss diagnosed could be diagnosed and and mife and miso prescribed for treatment.

**Response**: We thank the reviewer for asking us to clarify this. To be explicit, this criterion did not require a Z33.2 code or any other IAB-specific code. It required the complete absence of any pregnancy-loss diagnosis code, including the expanded SAB code set from Modification 4 anywhere in the pregnancy episode. Our reasoning was that guideline-concordant medical management of a diagnosed loss would ordinarily generate a corresponding diagnosis code at the treating encounter. The absence of any such code across the full pregnancy episode, combined with an early gestational-age dating claim and a prescription within 7 days, was interpreted as evidence favoring an undocumented IAB over an undocumented SAB. We agree this in an inference rather than direct evidence, and that some genuinely medically-managed SAB cases lacking diagnosis documentation could be misclassified as IAB under this criterion. In practice, this pathway affected only 3 previously unclassified pregnancies in our cohort (see Appendix Table 2), limiting its influence on overall estimates. We have clarified this logic in the Methods.

Line 211: This approach is designed to capture medication abortions that are managed entirely in the outpatient setting without a diagnosis code explicitly documenting the abortion (12).

**Response:** See above.

Line 193-201: What proportion of IAB and SAB without an outcome diagnosis code were those with outpatient mife/miso NDC and HCPCS code?
**Response**: We thank the reviewer for this question. We have utilized the data from Appendix Table 2 and the logic outlined in our methodology to break down these proportions. Under Modification 3, outpatient NDC and HCPCS claims for mifepristone and/or misoprostol were used to affirmatively classify pregnancies that lacked an explicit outcome diagnosis code (previously classified as “Unknown” in the Ailes et al. algorithm). As shown in Appendix Table 2, this specific clinical scenario applied to only 3 pregnancy episodes out of the total 68,084 IAB cases (<0.01%). The vast majority of IAB cases in our sample already carried explicit outcome codes. None of the SAB cases without an outcome diagnosis were identified via mifepristone and/or misoprostol claims. As detailed in Modification 2, the 68,531 pregnancies reclassified from “Unknown” to “SAB” were identified strictly based on a presentation of early gestational age (< 56 days) followed by a ≥60-day complete cessation of all obstetric claims. This specific cohort represents early pregnancy loss managed expectantly without medical or surgical intervention.

Line 211: I am not sure that reference 12 fully supports the proposed explanation. The manuscript suggests that medication abortions managed in outpatient settings without an abortion diagnosis code may have become more common following telehealth expansion post-Dobbs. However, the relationship between telehealth expansion and claims-based ascertainment is not entirely clear from the cited source. Additional information regarding the temporal stability of Z33.2 coding and telemedicine ascertainment in the dataset, mifepristone/misoprostol prescribing, and outcome classification over the study period would help readers evaluate this interpretation. Given that 94% of IABs were associated with Z33.2 codes, the observed temporal trends may warrant further discussion. That said, the practical impact of this reclassification decision appears relatively small. This seems to be in conflict with the logic presented in line 341.

**Response:** We have softened the language in lines 211-214 to allow the reader to make their own conclusion regarding our approach.

**Original Text**: This approach captures medication abortions that are managed entirely in the outpatient setting without a diagnosis code explicitly documenting the abortion, a pattern that may be increasingly common following the expansion of telehealth-based medication abortion access in the post-Dobbs period (12).

**New Text (lines 211-214**): This approach is designed to capture medication abortions that are managed entirely in the outpatient setting without a diagnosis code explicitly documenting the abortion (12).

Line 294: when comparing regions, I would keep the order of IAB vs SAB in parentheticals consistent (ie list IAB first in both to illustrate the stark geographic contrast)

**Response:** We have adjusted the orders of the parentheticals for clarity.

Line 341: I was uncertain whether the cited source directly supports the assertion that telemedicine abortion is undercaptured in insurance claims. The KFF resource primarily describes state-level variation in telehealth abortion access, whereas telehealth utilization and reimbursement more broadly increased substantially during and after the pandemic (10.1377/hlthaff.2018.05116), as did telehealth provision of medication abortion (e.g., WeCount reports).

It may therefore be more conservative to frame the finding as reflecting changes in the site and modality of abortion care delivery, which could influence claims-based ascertainment, rather than assuming reduced claims capture. For example, the 2021 decline may reflect pandemic-related disruptions in healthcare utilization and rapid changes in abortion care delivery. Additional discussion of temporal trends in telemedicine use, if available, would help support the proposed interpretation. Given the relatively short pre-pandemic observation period, it is also difficult to determine whether the 2021 decline and subsequent rebound represent meaningful departures from baseline variation.

**Response:** We have updated this reference to the Health Affairs article cited by the reviewer and agree that it better fits this claim. We have also softened the language.

**Origianl Text:**. In 2021, there was a decrease in the number of IAB identified which may represent a COVID-19 shift to alternative delivery for abortion care such as telemedicine access which is likely to be under-captured in insurance claims^19^.

**New Text (lines 342-346):** In 2021, there was a decrease in the number of IAB identified which may represent a COVID-19 shift to alternative delivery for abortion care such as telemedicine access which may be under-captured in insurance claims^19^.

Line 340-348: I appreciated the authors' discussion of potential post-Dobbs changes in outcome ascertainment. A few additional considerations may strengthen this section.
First, because the pre-period begins in 2019, it is difficult to determine whether the post-2022 increase in identified SAB represents a meaningful departure from longer-term trends.
Second, while the authors appropriately note that it is unclear whether the increase reflects a true biologic trend, several additional explanations may warrant consideration. These include expansion in insurance coverage and mixed healthcare utilization patterns that affect whether pregnancy losses generate billable encounters, follow-up care related to self-managed abortion, which increased following Dobbs, and changes in provider documentation or billing practices in response to institutional guidance or evolving legal considerations surrounding abortion care - perhaps this lead to more specific documentation of SAB to avoid legal scrutiny in mifepristone prescription.
More broadly, the observed increase in claims-identified SAB may reflect changes in healthcare-seeking behavior or claims-based ascertainment rather than changes in the underlying incidence of pregnancy loss. A brief discussion of these alternative mechanisms would strengthen the interpretation of the temporal trends.

**Response**: We agree with the above suggestions and have edited the manuscript language.

**Original T**ext: Vital statistics data beyond 2019 has not been published, so it is unknown if this is a true biologic trend^20^. Instead, the increase may reflect a post-*Dobbs* trend of patients seeking SAB care within healthcare settings that more commonly bill insurance due to access issues, or IAB being miscoded as SAB to avoid legal persecution. However, we did see a reciprocal decrease in IAB claims.

**New Text (Lines 347-352)**: Vital statistics data beyond 2019 has not been published, so it is unknown if this is a true biologic trend^20^. Instead, the observed increase in claims-identified SAB may reflect changes in healthcare seeking behavior or claims based ascertainment related to the post-Dobbs landscape, rather than a true change in rates of pregnancy loss. The observed increase in SAB could be due to an expansion in coverage, changes in provider documentation or billing practices in response to legal considerations surrounding abortion care, or mixed healthcare utilization patterns.

Line 347: The discussion of post-Dobbs "access issues" is interesting. Could the authors elaborate on the specific barriers they have in mind (e.g., geographic access, telemedicine availability, provider capacity, insurance-related barriers, or other factors)? Additional detail may help readers interpret the proposed explanation for the observed trends.

**Response:** We have added to the above response.

**New Text (Lines 355-357):** ...Following Dobbs, patients and providers may have been less likely to generate IAB claims due to geographic restrictions, lack of telemedicine availability or provider discomfort.

Reviewer #2: Fuerst et al. proposed an algorithm to identify spontaneous abortion (SAB) using administrative data. The core research question is possible ambiguity in identification of SAB with induced abortion (IAB) in available databases and established algorithms. Overall, this study offers a good example showing the complexity in the real-world in recording SAB and IAB. However, the sensitivity and specificity of the algorithm cannot be evaluated within the administrative database but needs to be assessed in a eMR system with both clinical records and insurance claims records.The key algorithm change proposed by this study is to move IAB prior to SAB. Authors provided several sensitivity analyses and clearly presented results. However, all “sensitivity” measure of the algorithm is not valid and should be presented as estimation under different assumptions/algorithms design.
Two improvements might be considered:
1. Use a complete eMR database/hospital eMR data to assess sensitivity & specificity.

**Response:** Unfortunately, we do not have access to a linked electronic medical record database to verify the true specificity and sensitivity of our algorithms' ability to identify and distinguish SAB from IAB. Of note, this is something we are working to validate but using a different and much smaller database. When we use the term ‘sensitivity’ to refer to a *sensitivity analysis* where we alter input variables to see if they significantly change our results and suggest major flaws in our identification algorithm.

2. If the formal validation is not possible, it will be the best to list detailed ICD/CPT codes of those pregnancies with different results. Table structure may be similar as in Appendix Table 3, will be the best to list the N by different scenarios. e.g. with columns code, description, Scenario A (A. SAB, F. IAB), Scenario B (A. IAB, F. SAB), ......

**Response:** Given the number of billable events within each pregnancy episode, listing all the codes for the pregnancy episodes that could be coded as SAB or IAB (depending on algorithm order) is simply not feasible for the scope of this paper. We appreciate this suggestion and this could be considered with future work using smaller databases. We are currently conducting other work to try and better identify clinical scenarios that may lead to coding ambiguity. However, given the size of the marketscan database, we cannot realistically present this data within the scope of this paper. Despite this limitation, we do not anticipate this would change our core findings.
